# Ferroptosis: a potential target for the treatment of atherosclerosis: Ferroptosis and atherosclerosis

**DOI:** 10.3724/abbs.2024016

**Published:** 2024-02-07

**Authors:** Chengyi Li, Ran Liu, Zhenyu Xiong, Xue Bao, Sijia Liang, Haotian Zeng, Wei Jin, Quan Gong, Lian Liu, Jiawei Guo

**Affiliations:** 1 School of Medicine Yangtze University Jingzhou 434020 China; 2 Department of Pharmacology Zhongshan School of Medicine Sun Yat-Sen University Guangzhou 510120 China; 3 Department of Gastroenterology Shenzhen People’s Hospital The Second Clinical Medical College Jinan University Shenzhen 518000 China; 4 Department of Second Ward of General Pediatrics Suizhou Central Hospital Hubei University of Medicine Suizhou 441300 China

**Keywords:** atherosclerosis, ferroptosis, Gpx4, lipid peroxidation

## Abstract

Atherosclerosis (AS), the main contributor to acute cardiovascular events, such as myocardial infarction and ischemic stroke, is characterized by necrotic core formation and plaque instability induced by cell death. The mechanisms of cell death in AS have recently been identified and elucidated. Ferroptosis, a novel iron-dependent form of cell death, has been proven to participate in atherosclerotic progression by increasing endothelial reactive oxygen species (ROS) levels and lipid peroxidation. Furthermore, accumulated intracellular iron activates various signaling pathways or risk factors for AS, such as abnormal lipid metabolism, oxidative stress, and inflammation, which can eventually lead to the disordered function of macrophages, vascular smooth muscle cells, and vascular endothelial cells. However, the molecular pathways through which ferroptosis affects AS development and progression are not entirely understood. This review systematically summarizes the interactions between AS and ferroptosis and provides a feasible approach for inhibiting AS progression from the perspective of ferroptosis.

## Introduction

Atherosclerosis (AS) is a chronic and progressive arterial disease primarily caused by interactions among vascular endothelial cell (EC) injury, lipid deposition, and the inflammatory response [1]. This disease is mainly associated with large- and medium-sized arteries, such as the coronary, carotid, and lower-extremity arteries [2]. AS is a prevalent cardiovascular disorder worldwide and is the primary contributor to adverse outcomes in individuals with cardiovascular and cerebrovascular conditions. AS is a chronic progressive disease that most frequently occurs in the elderly population. Although the incidence of AS has declined in some countries in recent decades, it remains the leading cause of death globally [3].

Lifestyle changes such as reducing carbohydrate and fat intake, engaging in regular physical activity, and avoiding smoking are essential components of a multifaceted approach to prevent AS progression [4]. However, lifestyle modifications are difficult to accomplish. Therefore, effective targets and safe therapeutic strategies are needed to reduce the incidence of AS [5].

Oxidative stress, characterized by an imbalance between the generation of reactive oxygen species (ROS) and the presence of antioxidants or free radical scavengers, plays a crucial role in AS [6]. A growing body of evidence suggests that ferroptosis is strongly associated with ROS generation, iron homeostasis, and lipid peroxidation induced by diverse physiological and pathological stressors in both humans and animal models [ 7– 9]. Ferroptosis, which is associated with iron and lipid metabolism, plays a pathological role in AS by linking it to oxidative stress.

Iron in blood was first identified in the 18th century; however, iron metabolism was not described until the late 1930s [ 9, 10]. Ferroptosis makes cells vulnerable to lipid peroxidation and iron, and through glutathione (GSH) synthesis, the cystine/glutamate antiporter, system Xc ^‒^, and glutathione peroxidase 4 (GPX4) protect metabolic pathways, including mitochondrial respiration, fatty acid metabolism, the mevalonate pathway, and (selenium) mercaptan metabolism [ 11, 12]. Ferroptosis is the most recently identified iron-dependent form of cell death and is driven by the inactivation of GPX4 and subsequent accumulation of lipid peroxides [13]. In addition, it is triggered by dysfunction of system Xc ^‒^ [14]. Genetic studies have provided compelling evidence that the synthesis of GSH, the activity of system Xc ^‒^, and the function of GPX4 collectively confer protection against cell death induced by various oxidative stress stimuli, particularly those that lead to thiol depletion [ 15– 17].

This review focuses on key advances in understanding the molecular mechanisms of ferroptosis, the link between ferroptosis and AS, and the application of ferroptosis-relevant therapeutic targets in AS.

## Key Molecular Mechanism of Ferroptosis

Cell death can be induced by various processes. Since ferroptosis was first described as a form of iron-dependent nonapoptotic cell death in 2012 [9], research on ferroptosis has grown exponentially, and research on its underlying mechanisms has made rapid progress. Most of these investigations have focused primarily on cellular metabolism and have revealed the close interplay between ferroptosis and metabolic cascades. Ferroptosis can be triggered via two distinct routes: an exogenous or transporter-dependent pathway and an endogenous or enzyme-regulated pathway [18]. Ferroptosis is primarily attributed to an imbalance in the redox status of pro- and antioxidant factors and is driven by the aberrant expression and activity of diverse redox-active enzymes involved in the generation or elimination of free radicals and lipid oxidation products [19]. Ferroptosis is a form of cell death orchestrated by iron-dependent phospholipid peroxidation [20]. Its regulation involves a myriad of cellular metabolic pathways, including redox homeostasis, iron metabolism, mitochondrial function, and the metabolism of amino acids, lipids, and carbohydrates, as well as various disease-associated signaling pathways [19]. The initiation and development of ferroptosis are linked to iron, acid, and lipid metabolism, particularly in cardiomyocytes [21]. The metabolic pathways potentially involved in ferroptosis and CVD are discussed below.

### Iron metabolism

**Figure 1:**
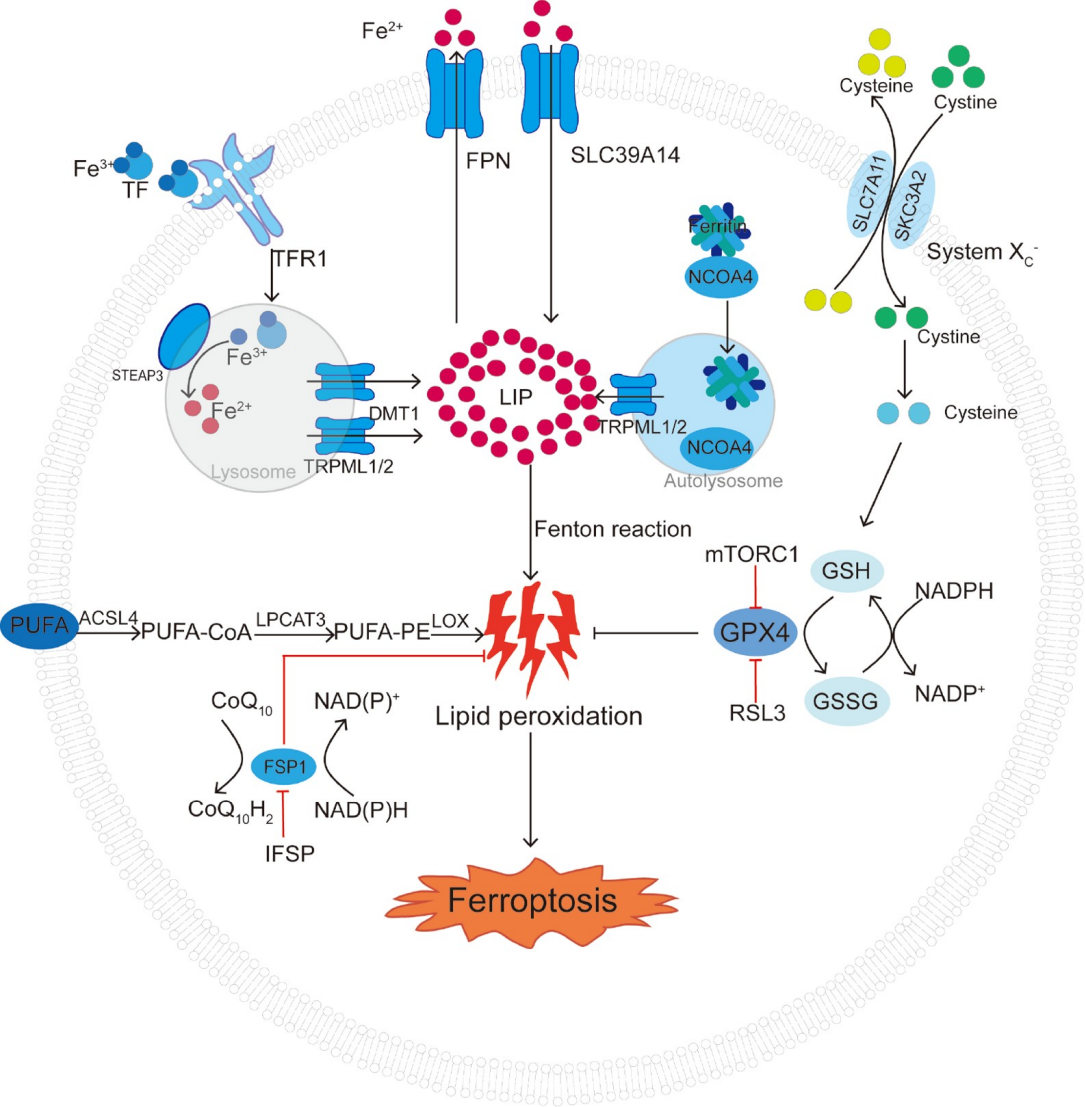
Figure 1 Basic mechanisms and regulatory pathways of ferroptosis Ferroptosis is related to intracellular free Fe2+ metabolism disorders or dysfunction of glutathione peroxidation and polyunsaturated fatty acid lipid peroxidation. This figure illustrates the basic process of ferroptosis and shows the inducers and inhibitors of related processes. The black arrows and red blunt lines represent the promotion and inhibition of ferroptosis, respectively. TF, transferrin; TFR1, transferrin receptor 1; STEAP3, six-transmembrane epithelial antigen of prostate 3; DMT1, divalent metal ion transporter 1; TRPML1/2, mucolipin TRP channel 1/2; FPN, ferroportin; SLC39A14, metal transporter protein; NCOA4, nuclear receptor coactivator 4; LIP, labile iron pool; PUFA, polyunsaturated fatty acid; ACSL4, acyl-CoA synthetase 4; LPCAT3, lysophosphatidylcholine acyltransferase 3; LOX, lysyl oxidase; IFSP, inhibitor ferroptosis suppressor protein; FSP1, ferroptosis suppressor protein 1; NADPH, nicotinamide adenine dinucleotide phosphate; CoQ10H2, ubiquinol; GSH, glutathione; GSSG, glutathione disulfide; GPX4, glutathione peroxidase 4; mTORC1, mechanistic target of rapamycin complex 1.

Systemic iron homeostasis is tightly regulated through iron uptake, recycling, and loss. Excess Fe directly induces ferroptosis. The body absorbs iron in the duodenum [ 22 , 23]. Cytosolic iron in intestinal cells can be stored as ferritin or exclusively exported into the plasma by the basolateral iron exporter, the iron transporter ferroportin (FPN) [24]. The absence or downregulation of FPN level is an important factor contributing to iron overload and ferroptosis [25]. Iron binds directly to transferrin (TF) and is subsequently transported to cells [26]. Before passing through the cell membrane, the iron reductase Cybrd1 (DcytB) is required to reduce nonheme ferric iron (Fe ^3+^) to ferrous iron (Fe ^2+^), which is absorbed by divalent metal transporter 1 (DMT1) [27]. Once divalent iron is absorbed by DMT1, it passes into intestinal cells and is transferred to different locations in the cell to meet the iron requirements of the cells and organelles [28]. Dietary iron exists mainly as Fe ^3+^, which can be reduced by iron reductases [29]. Iron toxicity originates from the Fenton reaction between Fe ^2+^ and Fe ^3+^ ( Figure 1), which results in the production of ROS that can damage lipids, proteins, and DNA, thereby causing ferroptosis. Some chemicals, such as malachite green, can bind to apotransferrin and alter iron transfer [30]. FPN is internalized by the hormonal peptide hepcidin and is subsequently degraded by lysosomes in iron-deficient cells, thereby preventing iron outflow into the extracellular matrix [31].

Ferritin can store up to 24 Fe atoms in a 4500-subunit macromolecular complex composed of light and heavy chains. Specific lysosomal degradation of ferritin releases iron and supplies the iron required by cells [32]. Since excess free iron can be toxic to cells, it is released from endosomes into unstable iron pools via DMT1 to avoid cytotoxicity [33]. This is an important physiological process of iron metabolism [34]. The absorption of iron within the intestinal tract is meticulously regulated and depends on the iron demands of the organism [ 34, 35]. Iron metabolism disorders can directly or indirectly impair macromolecules, including proteins, nucleic acids, and lipids, leading to cell damage or death [36].

### Lipid peroxidation

Lipid metabolism is also closely associated with ferroptosis [37]. Among the siderophores, all the pathways involve iron-dependent lipid ROS accumulation [38]. Ferritin phagocytosis occurs when ferritin interacts with other cells or molecules. Ferritin binds to a receptor on the cell surface and enters cells via phagocytosis [39]. Once inside the cell, ferritin is degraded by acidic lysosomes. The stored iron ions are then released, increasing cellular iron levels, leading to the accumulation of ROS and eventually cell death [40]. Although iron is an inorganic nutrient that is essential for cell proliferation, excess iron in the body produces Fe ^2+^ ( Figure 1), which participates in the Fenton reaction (Fe ^2+^+H _2_O _2_→Fe ^3+^+ ·OH+OH ^‒^) [ 41, 42]. This process generates hydroxyl radicals (·OH) that directly attack lipids, leading to peroxidation of polyunsaturated fatty acids (PUFAs), which further leads to ferroptosis [42]. Free iron levels in cells must be tightly regulated to prevent ROS production via the Fenton reaction [7]. The redox capacity of GSH is considered the main mechanism through which it reduces ROS levels [43]. GSH is a tripeptide composed of glutamic acid, cysteine, and glycine. GSH acts as an antioxidant and a substrate for GPX4 and is converted to oxidized GSH (GSSG) [38]. The loss of activity of the lipid-repairing enzyme GPX4 and the subsequent accumulation of lipid ROS, particularly lipid hydroperoxides, drive ferroptosis [44]. Most cellular cysteine residues are involved in biosynthetic processes that inhibit protein translation. Cellular cysteine and GSH exhibit concomitant protective effects against ferroptosis, thereby reinforcing their collaborative involvement in the modulation of cellular signaling pathways [45].

System Xc ^‒^ facilitates the absorption of cystine and is crucial for the production of GSH, activation of GPX4, and protection of cells from ferroptosis [9]. System Xc ^‒^ also plays a pivotal role in facilitating the uptake of cystine and is instrumental in the synthesis of GSH and activation of GPX4, thereby exerting a critical protective effect against ferroptosis [9]. This amino acid transporter is widely distributed in phospholipid bilayers and consists of a heterodimer composed of two subunits: solute carrier family member 7 member 11 (SLC7A11) and SLC3A2. Heterodimers are key components of the cellular antioxidant system [46]. Importantly, system Xc ^‒^ enables the bidirectional transport of cystine and glutamate across the cell membrane at a 1:1 ratio ( Figure 1). Notably, the metabolite Neu5Ac was recently found to promote ferroptosis in the vascular endothelium and aggravate atherosclerotic pathology by degrading SLC3A2 [47]. The absorbed cystine is enzymatically reduced in the cell to form cysteine, which plays a crucial role in GSH synthesis [9]. Additionally, P53 inhibits cystine uptake by downregulating SLC7A11 expression, which affects GPX4 activity. This inhibition results in reduced cellular antioxidant capacity and the accumulation of lipid ROS, ultimately leading to ferroptosis [ 48– 50]. RSL3 functions as a potent ferroptosis inducer by directly targeting and inhibiting GPX4. This inhibition diminishes cellular antioxidant capacity, resulting in the accumulation of ROS and ultimately culminating in ferroptosis [51].

During ferroptosis ( Figure 1), reduced GPX4 activity leads to catastrophic membrane rupture caused by iron-induced lipid peroxidation [52]. GPX4 uses GSH as a cofactor, with cysteine being the rate-limiting factor for GSH synthesis [53]. Systemic Xc ^‒^ inhibition leads to the depletion of GSH and impairs GPX4 activity, resulting in increased lipid peroxidation.

### Amino acid metabolism

Iron is involved in the synthesis of several important proteases and is an important component of human life [ 54– 56]. However, the mechanism underlying ferroptosis remains unclear. The failure of the GSH-dependent antioxidant defense system has been proposed to cause ferroptosis [ 57, 58]. Several investigations have identified various molecular constituents implicated in ferroptosis and revealed their intimate associations with cellular metabolism and redox pathways [20]. Ferroptosis is induced by two serum factors: the amino acid glutamine and the iron carrier protein transferrin [59]. A crucial aspect of ferroptosis is the suppression of glutamine catabolism [9]. System Xc ^‒^ is a disulfide-linked heterodimer and sodium-dependent cystine/glutamate exchange transporter protein composed of two subunits: a heavy chain (CD98hc, SLC3A2) and a light chain (XcT, SLC7A11) [60]. Extracellular cysteine is exchanged for intracellular glutamate at a 1:1 ratio via this transporter. System Xc ^‒^ imports cystine into cells and converts it into cysteine to synthesize GSH [61]. GSH deoxygenates PL-PUFA(PE)-OOH to PL-PUFA(PE)-OH with the help of GPX4 ( Figure 1), thereby protecting cells from ferroptosis. GSH is an important antioxidant and free radical scavenger. It is converted to GSSG in the presence of GPX4, resulting in the production of nontoxic compounds from toxic peroxides [62]. Hemin, an inducer of heme oxygenase-1 (HO-1), accelerates erastin-induced iron-dependent cell death [63]. The inhibition of system Xc ^-^ expression can render cells susceptible to ferroptosis [ 64, 65]. Iron can influence the metabolism of amino acids, particularly through metabolic pathways, including glutathione (GSH), which affects lipid metabolism. This disruption in the regulation of iron concentration can lead to excess iron levels, resulting in ferroptosis.

## Ferroptosis in the Pathogenesis of AS

**Figure 2:**
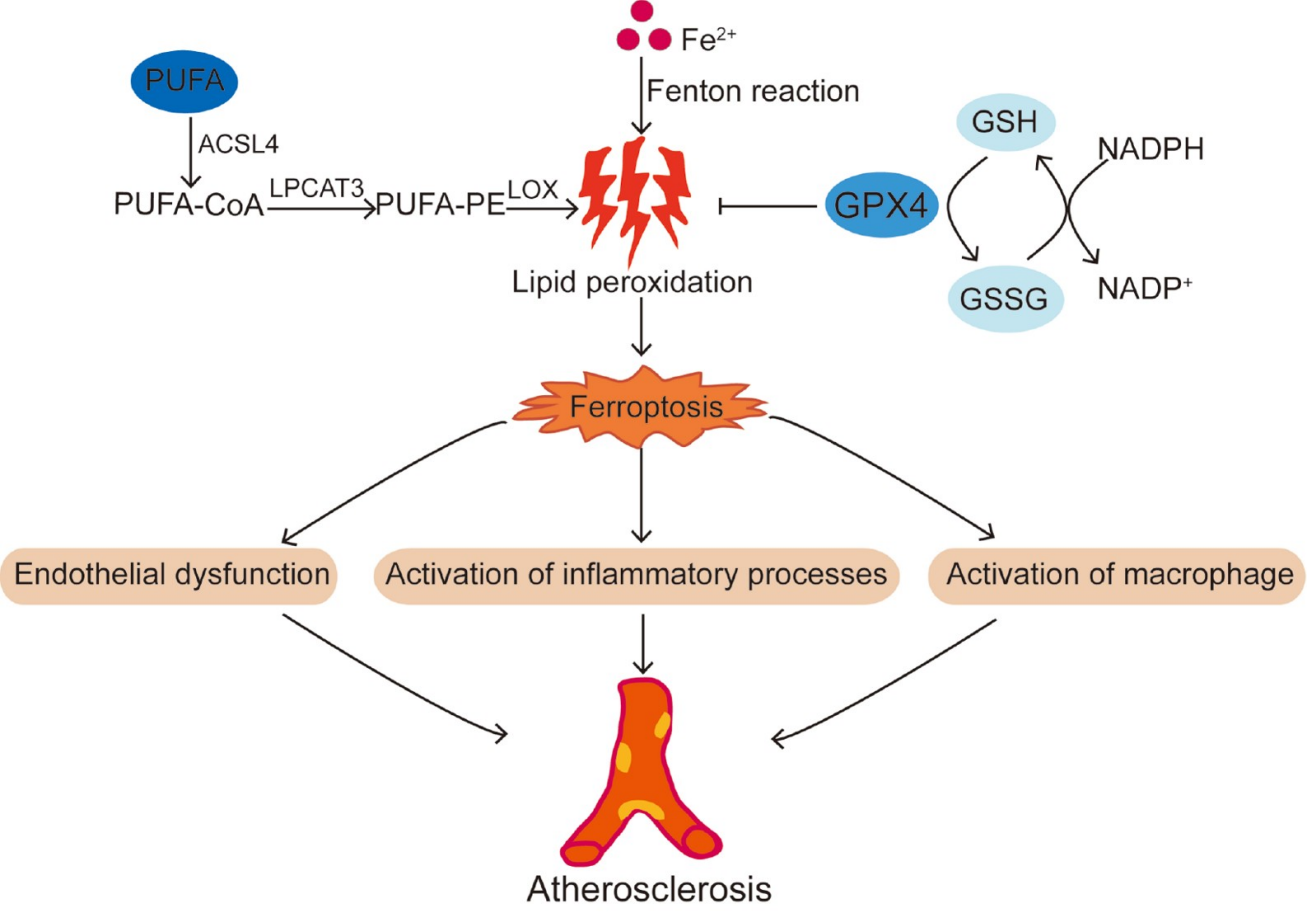
Figure 2 Schematic diagram of the progression of atherosclerosis caused by ferroptosis The occurrence of ferroptosis from most causes increases the risk of atherosclerosis. Ferroptosis leads to endothelial dysfunction, activation of inflammatory processes and activation of macrophages, which causes atherosclerosis. PUFA, polyunsaturated fatty acid; ACSL4, acyl-CoA synthetase 4; LPCAT3, lysophosphatidylcholine acyltransferase 3; LOX, lysyl oxidase; NADPH, nicotinamide adenine dinucleotide phosphate; GSH, glutathione; GSSG, glutathione disulfide.

AS is typically caused by endothelial dysfunction. Oxidized forms of low-density lipoprotein (LDL), which transport cholesterol in the blood, accumulate and lead to local inflammation and excess ROS production [ 66– 68]. ROS-induced inflammasome activation and lipid peroxidation are crucial features of AS [69]. Ferroptosis plays an important role in the pathogenesis of AS by linking oxidative stress, inflammation, and lipid metabolism [69]. Inflammation sites recruit monocytes that differentiate into macrophages [70]. Macrophages die after ingesting ox-LDL and provide positive feedback by recruiting more immune cells to areas of inflammation [ 71, 72]. This is also associated with the transdifferentiation of smooth muscle cells and fibrostromal hyperplasia [73]. Subsequently, atherosclerotic plaques form in the lining of the arteries, mainly due to inflammation [74]. The narrowing or blockage of blood vessels that results in CVD is caused by platelet aggregation, thrombosis, and rupture of unstable atherosclerotic plaques, all of which can lead to CVD. Ferroptosis is important in the pathophysiology of AS, as demonstrated in epidemiological studies and animal experiments [75]. Ferroptosis may also regulate the development of AS ( Figure 2) [75]. Thus, the suppression of ferroptosis may reduce AS by reducing lipid peroxidation and lipid dysfunction in aortic ECs [76]. Furthermore, the inhibition of ferroptosis may reduce AS. The genes and mechanisms linked to AS-associated ferroptosis are described in this section.

### Endothelial dysfunction associated with ferroptosis

Recent findings [77] have shown that ferroptosis is associated with EC death. For example, Qin *et al* . [78] reported that zinc oxide nanoparticles (ZnONPs) induce iron and lipid peroxidation in ECs in a dose- and time-dependent manner. The authors used the lipid and ROS scavenger ferrostatin-1 and the iron chelator deferoxamine to attenuate ZnONP-induced ferroptosis in ECs [79]. Ferroptosis is associated with endothelial dysfunction and is regulated by the p53-xCT-GSH axis in ECs [79]. Lysophosphatidylcholine (LPC) increases intracellular iron and lipid peroxide levels and causes mitochondrial atrophy in ECs, which can be reversed by astragaloside IV [80]. Thus, ferroptosis is an important mechanism by which ROS induce programmed cell death in ECs. Dysfunction of vascular ECs contributes to the development of AS [ 81, 82]. EC dysfunction and death lead to the release of inflammatory cytokines and the recruitment of monocytes, which initiate AS [83]. Modulation of ferroptosis in ECs accelerates the progression of atherosclerotic plaques.

Ferroptosis is associated with ox-LDL-induced lipid accumulation in endosomes. Lipid peroxidation in the inner leaflet of the plasma membrane may be important in ferroptosis [ 51, 84 ]. GPX4, which reduces lipid peroxides to lipid alcohols (L-OH), is a key regulatory factor of lipid peroxidation. GSH is a substrate of GPX4 [85]. The reduced availability and activity of GPX4 leads to the accumulation of membrane lipid peroxides, oxidative lipid damage, and subsequent ferroptosis [ 9, 51, 85 ]. Lipid peroxide concentrations are significantly greater in patients with AS (in both coronary and peripheral arteries) than in controls [86]. A reduction in the levels of lipoprotein (a) and its associated oxidized lipids is being investigated as an alternative treatment strategy for AS [46]. The inhibition of ferroptosis may alleviate AS by attenuating lipid peroxidation and endothelial dysfunction in aortic ECs. ACSL4 determines the vulnerability of a cell to ferroptosis by influencing lipid composition [87]. Protein kinase C βII (PKCβII) is a sensor of lipid peroxidation. The lipid peroxidation-PKCβII-ACSL4 positive feedback axis may provide potential targets for the treatment of ferroptosis-associated diseases [75]. RSL3 also induces ferroptosis, and the antioxidant defense enzyme RSL3 has been identified as a direct drug target of RSL3 using a chemoproteomic approach [51]. Reduced cellular iron intake or chelation prevents the generation of ROS, which is associated with RSL-induced cell death. Thus, ferroptosis is the term for cell death caused by the buildup of iron-dependent cellular ROS, which ultimately results in the disruption of the cellular redox equilibrium [88]. Moreover, ferroptosis inducers and inactivation of dihydroorotate dehydrogenase work together to induce mitochondrial lipid peroxidation [89]. A recent study reported that vitamin D receptors inhibit ferroptosis by regulating the AMP-activated protein kinase signaling pathway and adrenomedullin transcription, thereby alleviating lipid deposition *in vivo* and *in vitro* [90].

The development of AS is often attributed to the multilayered intima. However, atherosclerotic plaques are the result of many mechanisms involving both resident and invasive inflammatory cells [ 91, 92]. Ferroptosis suppression attenuates AS by decreasing lipid peroxidation and endothelial dysfunction in aortic ECs of mice [76]. Recently, it has been reported that aluminium exposure promotes atherosclerosis by inhibiting paraoxonase-1 activity and inducing endothelial dysfunction and adhesion molecule expression [93].

### Activation of inflammatory processes associated with ferroptosis

The main mechanism by which ferroptosis exerts immunological effects is the death of leukocyte subsets and the corresponding loss of immune function. For example, ferroptosis induces lipid peroxidation in T cells and promotes viral and parasitic infections [94]. In contrast to the widely held assumption that polymorphonuclear neutrophils have minimal consequences in AS, and evidence indicates that these cells play an important but unrecognized role in AS development [95]. The leukocyte count is also positively correlated with coronary artery disease severity [96].

DAMP signals are released and activated in response to different forms of cell death, triggering distinct immunological and inflammatory responses. Ferroptosis is a type of inflammatory cell death associated with the production of lipid oxidation products or DAMPs [ *e*. *g*., high-mobility group box 1 (HMGB1)] following tissue damage or cancer treatment. For example, in aging and chronic diseases, the lipid peroxidation product 4-hydroxynonenal is a proinflammatory mediator that triggers the nuclear factor-kappa B (NF-κB) signaling pathway [97]. Released by ferroptotic cells, HMGB1 is an archetypal DAMP implicated in multiple forms of cell death [98]. HMGB1 subsequently initiates an inflammatory response in peripheral macrophages through the activation of the NF-κB-activating advanced glycosylation end-product-specific pattern recognition receptor in innate immunity [99].

Through the RAS-c Jun N-terminal kinase (JNK)/p38 pathway, HMGB1 also regulates ferroptosis. HMGB1 has also been identified as a potential target for therapeutic intervention in leukemia [100]. However, there are few reports on whether HMGB1 regulates AS through the RAS-JNK/p38 pathway, which may constitute a direction for further research [100]. Targeting lipid metabolism-related DAMP signaling may be a promising strategy for treating inflammatory diseases related to the damage caused by ferroptosis.

Although some observations are suggestive, the causal role of erythrocytes in the development and progression of AS has not been determined, partly because of the simultaneous infiltration and activation of inflammatory cells that promote AS [ 101– 103]. Furthermore, the early transcriptional response caused by ox-LDL-containing immune complexes (ox-LDL-ICs) may be the basis for cytoprotection and promotion of inflammation [104]. The cross-linking of FcγRs appears to be the cause of most transcriptional responses to ox-LDL-ICs. These findings further reinforce the hypothesis that ox-LDL and ox-LDL-ICs induce different inflammatory responses and play different roles in AS [104].

### Activation of macrophages associated with ferroptosis

In the pathological model of AS, plaques contain several phenotypic subgroups of macrophages, which behave differently [ 105, 106]. AS-related ferroptosis may be associated with certain macrophage subtypes [107]. Macrophages affect the development of atherosclerotic plaques, and the M1 (inflammatory)/M2 (anti-inflammatory) macrophage balance is thought to affect disease progression [108].

The migration of macrophages from sites of inflammation can slow or stop plaque growth, allowing the material to leave the plaque. However, this depends on the presence of living foam cells deep within the plaque [109]. The phagocytosis of red blood cells by plaque macrophages promotes ferroptosis [110]. Olfactory receptor 2, located in vascular macrophages, drives AS development through the production of NOD-like receptor family pyrin domain-containing 3 (NLRP3)-dependent interleukin-1 [111]. M1, M2, and M4 macrophages are found in atherosclerotic plaques [112]. Within the plaque, macrophages are exposed to cytokines, chemokines, and bioactive lipids, such as cholesterol and oxidized phospholipids [112]. *In vivo*, M1 macrophages (compared to M2 cells) exhibit greater resistance to pharmacologically induced ferroptosis. This resistance is reduced in cells deficient in induced NO synthase under proinflammatory conditions caused by brain injury or in the tumor microenvironment [113]. The use of Fe _3_O _4_-SAS@PLT platelet membrane-camouflaged magnetic nanoparticles is a novel approach for enhancing iron toxicity and mild immunogenicity. This approach effectively changes macrophages from an M2 immunosuppressive phenotype to an M1 antitumor phenotype [114]. By increasing p300/CREB binding protein acetyltransferase activity and promoting p53 acetylation, the high ROS levels induced by iron overload polarize macrophages toward the M1 subtype [115]. In contrast, M2-polarized macrophage activation results in the production of neurotrophic factors [116] and the release of anti-inflammatory cytokines, such as interleukin-10, which have anti-inflammatory effects [ 117, 118 ].

Macrophages that take up cholesterol form foam cells that are deposited under the inner layer of the artery, eventually leading to AS [119]. By expressing cytokines and other factors, such as transforming growth factor-β1 (TGF-β1), macrophages can acquire different functional phenotypes and promote the osteogenic differentiation, chondrogenic differentiation, and angiogenesis of mesenchymal stem cells. HLF is regulated by TGF-β1 [120]. HLF transactivates gamma-glutamyltransferase 1 (GGT1) to enhance iron toxicity. GGT1 catalyzes the cleavage of extracellular GSH to supply cysteine for intracellular GSH production [121]. ALOX5 and neutrophil cytosolic factor 2 (NCF2) may be involved in the formation of necrotic cores in AS by regulating macrophage ferroptosis [30]. Changing the structure of macrophages by using certain substances can also affect the formation of foam cells and AS. For example, chondroitin sulfate N-acetylgalactosaminyltransferase-2 affects foam cell formation and AS by modifying the glycosaminoglycan chain [122]. In addition, iron loading exacerbates AS progression by enhancing glycolysis in macrophages [123]. Hepcidin increases the intracellular iron concentration in macrophages by inhibiting iron efflux, leading to ferroptosis and exacerbating inflammation and plaque development [124].

## Molecular Cross-talk between Ferroptosis and Other Types of Cell Death

Ferroptosis and other types of cell death, such as apoptosis, pyroptosis, necroptosis, autophagy, and cytoproptosis, involve molecular interactions involved in the occurrence and development of atherosclerosis. The molecular pathways of atherosclerosis are linked to these types of cell death in several ways.

### Apoptosis

Apoptosis, a type of programmed cell death, is usually triggered by internal or external signals and is the main type of cell death that occurs under homeostatic conditions, although there are many other types of cell death [125]. Iron ions in cells can promote an increase in oxidative stress-generated oxygen free radicals, thereby triggering the apoptotic signaling pathway [126]. Iron overload may lead to impaired mitochondrial function, which plays an important role in apoptosis [127].

Apoptosis is a key factor in atherosclerosis that influences plaque stability and accelerates disease progression. An increasing number of studies have suggested that the proinflammatory microenvironment of plaques, which is characterized by impaired apoptotic cell clearance, plays an important role in persistent inflammation [128]. Apoptosis affects VSMCs, macrophages, and endothelial cells and promotes plaque growth, inflammation, and thrombogenicity [129]. Atherosclerosis advances owing to an imbalance in the clearance of apoptotic cells as well as the effects of oxidative stress, inflammation, and ox-LDL [130]. In atherosclerosis, apoptosis may play a role in arterial endothelial and smooth muscle cells, leading to plaque formation and arterial stenosis [131]. It is crucial to comprehend these mechanisms to prevent and treat atherosclerosis, as they could lead to possible targets for management and therapeutic interventions.

### Pyroptosis

Pyroptosis is a form of cell death triggered by excessive heat inside cells. Iron has been shown to activate ROS signaling through the new Tom20-Bax-caspase-3-gasdermin D (GSDMD) pathway, thereby increasing cell death in melanoma [132]. There is a close relationship between the onset of focal cell death and AS. The integrity of the vascular endothelium is compromised during the initial phases of AS development due to risk factors such as hyperlipidaemia and oxidative stress that induce EC damage, which in turn causes the secretion of cellular inflammatory factors, leading to EC pyroptosis [ 133, 134]. Pyroptosis of VSMCs leads to further inflammation of blood vessels, exacerbates plaque instability, and promotes atherosclerosis progression [135]. The later stage of AS, known as AS plaque rupture, can occur when macrophage pyroptosis is sustained, during which copious amounts of inflammatory mediators are released and the inflammatory response is exacerbated *in vivo* [ 136, 137].

### Necroptosis

Necroptosis is a caspase-independent programmed cell death process [138]. Numerous signals typically initiate the necroptotic apoptotic pathway, which frequently results in the extravasation of cellular contents and additional activation of specific signaling pathways, leading to mixed lineage kinase domain-like pseudokinase (MLKL) phosphorylation, which converts the plasma membrane into oligomers and translocates them to the cell membrane. This positive feedback encourages cell rupture and the release of cell contents, initiating a chain of inflammatory reactions [139]. In addition, the presence of MLKL and activated major pro-necrosis factor 3 (RIP3) in atherosclerotic plaques indicate that necrosis is involved in the pathological progression of atherosclerosis [140] .

Although necroptosis is a different type of cell death from ferroptosis, there is evidence from several structural, functional, and mechanistic perspectives suggesting that these processes interact. The three positive factors of necroptosis are cysteine, HSP90, and the mitochondrial permeability transition pore (MPTP), whereas cysteine decreases ferroptosis by encouraging GSH synthesis and opening of HSP90 and MPTP to accelerate ferroptosis [141].

### Autophagy

Autophagy involves the degradation of proteins and organelles [142]. There is an interconnection between autophagy and ferroptosis, and autophagy can activate ferroptosis [ 143, 144]. The degradation of ferritin by autophagy increases the concentration of free iron in cells, resulting in ferroptosis [145]. Lipid autophagy promotes RSL3-induced lipid peroxidation and ferroptosis [146].

Autophagy slows the development of atherosclerosis by removing harmful substances from cells and protecting them from damage caused by oxidative stress and inflammation. Autophagy may also be involved in the pathogenesis of atherosclerosis. For example, aberrant activation of autophagy may lead to disturbances in intracellular lipid metabolism and increased inflammatory responses, thereby promoting atherosclerosis [ 130, 147]. The stress response and phenotypic transformation of VSMCs involve autophagy, which is generally a protective factor against atherosclerosis. Autophagy in problematic VSMCs further accelerates stress-induced premature aging and exacerbates the pathology of atherosclerosis [130].

### Cuproptosis

Cuproptosis, a new type of cell death, is caused by copper ions that selectively bind to lipoylated tricarboxylic acid cycle proteins. This leads to proteotoxic stress, which oligomerizes lipoylated proteins in a Cu-dependent manner, eventually causing cell death [148]. Cu plays an unanticipated role in enhancing iron-dependent cell death by activating macroautophagy/autophagic degradation of GPX4 [149].

According to recent studies, Cu ions may slow the initiation and progression of atherosclerosis by encouraging the formation of vascular smooth muscle cells and blocking pathways associated with inflammation [ 150, 151]. The serum Cu concentration is closely associated with atherosclerotic mortality [152]. However, the elemental copper content may vary with the severity of atherosclerotic lesions [153]. Furthermore, the role of copper differed between the groups, and these variations should be considered when administering copper for therapeutic treatment.

There are strong correlations between ferroptosis and the molecular mechanisms underlying apoptosis, pyroptosis, necroptosis, autophagy, and apoptosis. Focusing on common processes that occur during atherosclerosis, including ferroptosis and other modes of death, may provide new perspectives for disease prevention and treatment. Therefore, studying ferroptosis is highly important for further understanding the intermolecular mechanisms involved in cell death.

## Ferroptosis Is an Important Potential Treatment Target in AS

Ferroptosis is an important potential therapeutic target for the treatment and prevention of atherosclerosis. This section discusses the targets and summarizes some ongoing drugs ( Table 1) that regulate ferroptosis in AS.

**Table:** **Table 1** List of drugs that can regulate ferroptosis in AS

Drug	Target	Mechanism	Ref.
DiDang decoction	Upregulate GPX4	Improve mitochondrial function	[154]
Hydroxysafflor yellow A	Regulate SLC7A11 expression	Reduce atherosclerotic plaque formation	[155]
Sulforaphane and EPI-742	Regulate NRF2	Regulate lipid peroxidation	[156]
Vitamin E	Regulate NRF2	Regulate iron homeostasis	[157]
Micheliolide	Regulate NRF2	Regulate ferroptosis in macrophages	[158]
MI-2	Inhibit MALT1	Inhibit ferroptosis of vascular SMCs	[159]
Icariin	Promote autophagy	Inhibit ferroptosis	[160]

### Iron as a target and related drug

In 1981, the “iron hypothesis” was suggested. According to this hypothesis, increased iron stores can trigger cardiovascular diseases, whereas iron deficiency can prevent AS [ 161, 162 ]. Large deposits of iron in the middle layer of arteries are associated with plaque formation, oxidative stress, and vascular dysfunction [124]. Nontransferrin-bound iron (NTBI) acts at different levels in AS, modifying the serum and vascular microenvironment in a proatherogenic and proinflammatory manner; affecting vascular cell function and survival; promoting foam cell formation; and inducing angiogenesis, calcification, and plaque destabilization [163]. Iron overload or increased NTBI exacerbates AS in mice by promoting vascular dysfunction. The NTLI has been identified as a risk factor and therapeutic target for AS [164]. Gal-3 and vascular cell adhesion molecule 1, the two main factors involved in AS development, exhibit decreased expression levels after consumption of an iron-deficient diet [165]. In contrast, iron chelators or iron intake restriction may delay the development of atherosclerosis in ApoE ^−/−^ mice [124].

### GPX4 as a target and related drug

Accumulating evidence indicates that GPX4 is a key regulator of ferroptosis [ 166, 167]. GPX4 overexpression alleviates ferroptosis in AS by reducing lipid peroxidation [167]. In contrast, *GPX4* knockout inhibits bubbling in mouse bone marrow-derived macrophages by regulating ABCA1, ATP-binding cassette subfamily G member 1 (ABCG1), class A macrophage scavenger receptor (SR-A), and lectin-like ox-LDL receptor-1 (LOX-1) [168]. Further studies showed that the long noncoding RNA MRGPRF-6:1 inhibits GPX4 and exacerbates ferroptosis in macrophages [169]. The traditional Qing-Xin-Jie-Yu Granule prescription inhibits ferroptosis in atherosclerotic mice by upregulating GPX4/xCT level in aortic tissue [166]. The DiDang Decoction medicinal formulation activates the hypoxia-inducible factor-1 (HIF-1) signaling pathway and upregulates GPX4 level to inhibit atherosclerosis-associated ferroptosis [154]. These findings indicate that GPX4 may be a novel target for the treatment of atherosclerosis via the regulation of ferroptosis.

### FSP1 as a target and related drug

FSP1 and GPX4 constitute two major parallel ferroptosis defense systems. Inhibition of FSP1 results in effective ferroptosis [170]. Suppression of ferroptosis by FSP1 is mediated by ubiquinone, also known as coenzyme Q10 (CoQ10), which traps lipid peroxyl radicals that mediate lipid peroxidation, whereas FSP1 catalyzes the regeneration of CoQ10 using NAD(P)H. Although further experimental evidence is needed, the fact that FSP1 inhibitors promote macrophage infiltration suggests that FSP1 is a potential candidate for controlling AS through macrophages [171].

### SLC7A11 as a target and related drug

Increasing evidence indicates that ferroptosis regulates macrophage foaming [ 172, 173]. P53 suppresses SLC7A11 expression, which decreases cystine uptake and renders cells more susceptible to ferroptosis. An essential component of the cystine/glutamate antiporter is SLC7A11 [ 48, 174 , 175]. NF-κB inhibitors can override the regulation of the hepcidin/FPN/SLC7A11 axis by certain injury factors, thereby inhibiting ferritin formation in macrophages [ 173, 176]. In the aortic ECs of mice with type 2 diabetes mellitus and atherosclerosis, hydroxysafflor yellow A inhibits ferroptosis and atherosclerotic plaque formation by regulating the expression of SLC7A11 [155]. SLC7A11 has been suggested to be a potential therapeutic target for controlling macrophage expression in atherosclerosis.

### JAK signalling pathway as a target and related drugs

Among the common genetic variants that cause clonal hematopoiesis, the JAK2V617F (JAK2VF) mutation increases the JAK-signal transducer and activator of transcription signaling and occurs at a younger age, leading to the greatest risk of premature CHD [177]. Abnormalities in red blood cell quantity and quality are caused by the expression of Jak2VF, which exacerbates AS. The JAK signaling pathway is a potential therapeutic target for lowering the risk of atherosclerosis.

### p38 as a target and related drugs

Ionizing radiation can cause lipid metabolism disorders, leading to atherosclerotic disease [178], and high doses of ionizing radiation accelerate plaque formation and aggravate atherosclerosis progression through the upregulation of p38/nuclear receptor coactivator 4-mediated ferritinophagy [178].

JNK and p38 inhibitors are associated with apoptosis and reverse heteronemin-induced cell death [179]. The selective inhibitor targeting p38α effectively hinders the activation of the mitogen-activated protein kinase (MAPK) pathway and the release of pro-inflammatory cytokines within lamina propria mononuclear cells. [180]. In addition, a recent study revealed that transaldolase inhibitss p38 mitogen-activated protein kinase (MAPK) signaling and CD36-mediated cholesterol uptake by upregulating GSH, ultimately inhibiting macrophage foaming and atherosclerosis [181]. Orai1-dependent entry of calcium ions (Ca ^2+^) promotes atherogenesis, possibly by decreasing apoptosis signal-regulating kinase 1 or inhibiting its downstream effectors JNK and p38 MAPK, thus reducing scavenger receptor A expression level and promoting foam cell formation and vascular inflammation, indicating that the Orai1 Ca ^2+^ channel is a potential therapeutic target for AS [182]. ClC-3 inhibits the expressions of scavenger receptors and the uptake of ox-LDL through the JNK/p38/MAPK signaling pathway, preventing macrophage foaming and significantly reducing atherosclerotic plaque formation [183].

### Nuclear factor erythroid 2-related factor 2 (NRF2) as a target and related drug

NRF2 is a key antioxidant molecule [184]. Several studies have indicated that NRF2 is involved in the regulation of cellular ferroptosis [ 172, 185 , 186]. A recent study revealed that estrogen inhibits oxidation and ferroptosis through the NRF2/GPX4 pathway, thereby alleviating the pathological process of AS [172]. In contrast, estrogen deficiency induces ferroptosis and exacerbates the pathological process of AS [172]. Heme oxidase 1 (HO-1) targets NRF2 and regulates iron overload and ferroptosis [185]. The NRF2-KEAP1 axis controls inflammation and preserves redox, metabolic, and protein homeostasis to regulate ferroptosis [186]. NRF2 is a major factor involved in inducing cell survival under GSH depletion, and the role of l-butylthionine-(S,R)-sulfoxide (BSO) as a chemical sensitizer may be enhanced by inhibiting Nrf2 [187]. BSO induces GSH depletion; however, its role in ferroptosis and GSH activity has not been determined [188]. The use of sulforaphane and EPI-742 clarified the processes of lipid peroxidation and iron-dependent cell death in ferroptosis via the regulation of NRF2 [156]. This study highlights the potential of targeting NRF2-mediated ferroptosis as a treatment strategy for neurodegenerative illnesses, such as Friedreich′s ataxia [156]. Vitamin E supplementation controls iron homeostasis by inhibiting NRF2-mediated iron-responsive gene expression and increasing iron efflux through FPN in the liver [157]. Micheliolide, an aesquiterpene lactone, inhibits atherosclerosis by activating the NRF2 pathway to inhibit ferroptosis in macrophages [158]. Recently, single-cell transcriptomics revealed that, compared with control aortic macrophages, aortic macrophages from *Nrf2*-knockout mice exhibit differential changes in subtype-specific transcriptomes associated with inflammation, iron homeostasis, cell damage, and ferroptosis pathways [189]. Collectively, these findings suggest that NRF2 is a promising therapeutic target for the treatment of AS-related diseases.

### Other targets and drugs that can regulate ferroptosis in AS

Ferritin is composed of light and heavy chain subunits [190]. The human ferritin heavy chain can alleviate the pathological process of AS by inhibiting ferroptosis in the aortas of *ApoE*-knockout mice [191]. It is anticipated that the ferritin heavy chain is a potential target for the prevention of ferroptosis in AS patients.

An increasing number of studies have shown that microRNAs (miRNAs) play roles in AS development and progression [192]. A potential therapeutic strategy for AS involves blocking exosome-mediated transfer of miR-155 between the two cell types [193]. The delivery of miR-126-3p to ECs reduces the proliferation of vascular smooth muscle cells and inhibits neointima formation by inhibiting LRP6 [194]. Nicotine-induced exosomal miR-21-3p may accelerate AS development by enhancing vascular smooth muscle cell migration and proliferation via its action on phosphatase and tensin homologues [195]. In addition, MI-2, a specific chemical inhibitor, significantly mitigates endarteriopathies and atherosclerosis in ApoE mice by inhibiting the ferroptosis of vascular SMCs induced by mucosa-associated lymphoid tissue lymphoma translocation protein 1 (MALT1) [159] .

Endothelial Bach1 deficiency, reduced turbulent flow, or a Western-type diet induces atherosclerotic lesions, increases plaque macrophage counts, increases the expressions of endothelial adhesion molecules, including intercellular adhesion molecule 1 and vascular cell adhesion protein 1, and increases plasma tumor necrosis factor-α and interleukin-1 beta levels in mice with AS [21]. Thus, BACH1 is a potential novel therapeutic target for AS [196] .

Human and mouse plasma contain octanal, a product of lipid peroxidation, at sufficient concentrations to activate olfactory receptor 2 (Olfr2) and human olfactory receptor 6A2 (OR6A2). Increased octanal level exacerbates AS, whereas targeting Olfr2 in mice significantly reduces the formation of atherosclerotic plaques. These findings suggest that OR6A2 inhibition is a promising strategy for the prevention and treatment of AS [111].

Icariin is a bioactive compound with both antioxidant and anti-inflammatory properties. Icariin inhibits ferroptosis and alleviates atherosclerosis by promoting autophagy in ApoE mice [160] .

These findings provide new insights into the treatment of AS. These genes have been proposed to be potential therapeutic targets for controlling atherosclerosis through ferroptosis-related mechanisms.

## Conclusions and Prospects

The predominant features of ferroptosis are the disruption of iron homeostasis and the accumulation of lipid peroxides in conjunction with fatty acid synthesis, which are closely associated with AS. Therefore, ferroptosis may be a novel therapeutic target for the treatment of AS. This review describes the relationship between ferroptosis and the occurrence of AS and the molecular mechanism by which ferroptosis promotes the development of AS. The roles of transcription factors and signaling molecules in the development of ferroptosis are summarized in Table 1, and potential ferroptosis-related targets for the treatment of AS are presented. Prevention of AS, a critical initiating factor in the development of cardiovascular and cerebrovascular complications, including myocardial and cerebral infarction, is one of the greatest medical challenges worldwide. Ferroptosis plays an important role in the development of several systemic cardiovascular diseases. Although some animal models have provided evidence that ferroptosis may be a therapeutic target in AS, further *in vivo* experiments and clinical studies are needed. Further research on ferroptosis will deepen the understanding of AS pathogenesis and lead to improved clinical treatments.

However, the underlying mechanisms of AS pathogenesis have not been fully elucidated, and further research is needed. This review provides insights into the role of ferroptosis in AS pathogenesis. The identification of molecular events and effective drugs that inhibit ferroptosis is critical for treating AS.
